# Hidden Harlequin syndrome in neonatal and pediatric VA-ECMO

**DOI:** 10.1186/s13054-022-04017-w

**Published:** 2022-05-20

**Authors:** Yael Levy, Julie Starck, Anne-Lise Mary, Yohan Soreze, Sandrine Jean, Bernard Kreitmann, Pierre-Louis Léger, Jerome Rambaud

**Affiliations:** 1 Pediatric and Neonatal Intensive Care Unit, Armand-Trousseau Hospital, APHP, 26 avenue du Dr Arnold Netter, 75012 Paris, France; 2 Sorbonne University, Paris, France; 3 INSERM, IMRB, Univ Paris Est Créteil, 94010 Créteil, France; 4 Cardiac Surgery, Bordeaux University, Bordeaux, France

Extracorporeal membrane oxygenation (ECMO) is frequently used in critically ill children and, even if the reported results are good, it remains a challenge encompassing numerous risks. There are two types of ECMO: veno-arterial ECMO (VA-ECMO) which can be used for heart and lung support, while veno-venous (VV-ECMO) is used for lung support only. Indications for VA-ECMO, even if they must be carefully considered, peculiarly if respiratory failure is predominant, remain frequent in infants because there are several specific problems with the use of VV-ECMO. Various types of perforations have been reported with the use of a dual-lumen cannula in VV-ECMO [1]. In a survey of U.K ECMO centers, these complications have been cited as the main reason to favor VA-ECMO over VV-ECMO in infants with respiratory failure only [2]. Jugulo-femoral VV-ECMO should be avoided due to small femoral veins in infants and adequate ECMO flows cannot be achieved [3].

On bifemoral VA-ECMO in the adult population, when left ventricle (LV) recovery coexists with respiratory failure, an antegrade ejection by the LV of relatively deoxygenated blood hinders the retrograde oxygenated blood flow of the VA-ECMO in the aortic arch resulting in cerebral hypoxemia. This has been described as Harlequin syndrome (HS) [4]. In adults, this syndrome is detected by arterial blood gas and pulsatile saturation (SpO2) which decrease in the right arm but stay normal in the lower limb. In infants, the neck access for peripheral cannulation is commonly used. The drainage and infusion cannulas are introduced through the right internal jugular vein and the right carotid artery, respectively. In the literature, the HS has not been previously described in infants to our knowledge. However, in all infants on VA-ECMO, the LV continues to eject more or less “native” blood flow through the aortic valve. This is in opposition to the VA-ECMO blood flow in a point called “mixing-point,” located somewhere between the aortic valve and the brachiocephalic trunk [4]. If the cardiac native flow is insufficient (e.g., severe to moderate LV dysfunction or a very high afterload) and coexists with respiratory failure, the “mixing-point” will be localized into the ascending aorta, the detection of desaturated blood ejection from the LV may be hidden and the coronaries arteries will be the only arterial vessels perfused by this deoxygenated blood flow causing an “invisible” HS (Fig. 1A and B). This “invisible” HS could evolve to a secondary LV dysfunction explained by the desaturated coronary blood flow and the VA-ECMO afterload. Moreover, the reduction in the left ventricular preload by a large interatrial communication or the presence of LV dysfunction may be so important that the aortic valve remains closed, VA-ECMO then provides full circulatory support, no HS exists and the coronaries arteries are well oxygenated (Fig. 1C). In infants, the HS becomes “visible” if the LV function is mildly impaired or subnormal so that the “mixing-point” is close to the brachiocephalic trunk. Thus, the blood flows coming from VA-ECMO and the LV are mixed: SpO2 decreases in the descending aorta and allows easier detection of the HS (Fig. 1D, E and Additional file 1: Table S1 and Additional file 2: Table S2).

**Fig. 1:**
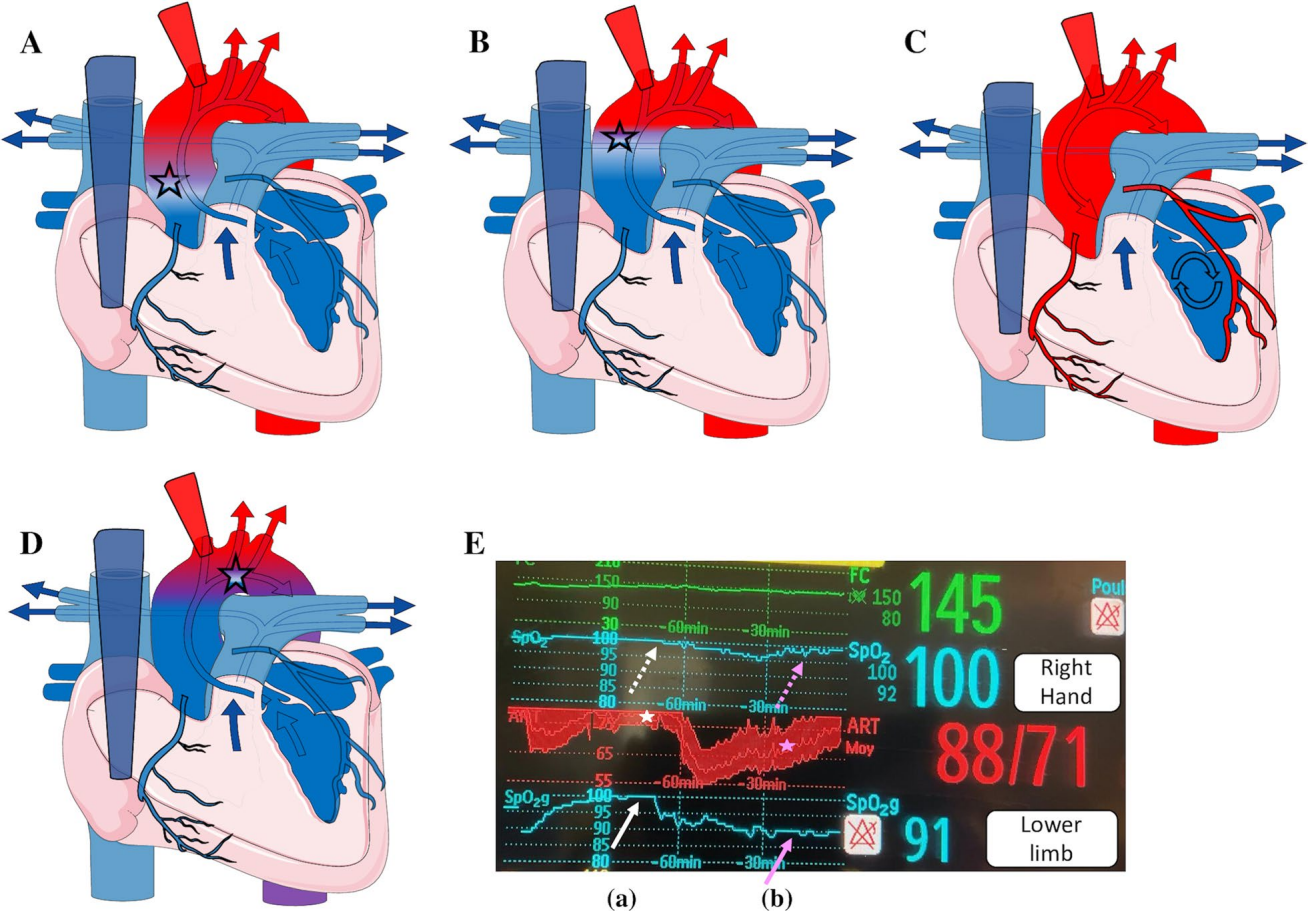
The Harlequin Syndrome during VA-ECMO in neonates and children with severe acute respiratory distress syndrome. In severe (**A**) to moderate (**B**) LV dysfunction, the “mixing” point is in the middle of ascending aorta. In these two situations, the Harlequin syndrome is invisible: the four limbs are fully oxygenated but the coronary arteries are not. **C** In profound left ventricle (LV) dysfunction there is no LV ejection and no mixing. The Harlequin syndrome is not present, coronary arteries are well oxygenated. D. In the subnormal LV recovery, the “mixing” point is close to the beginning of the brachio-cephalic trunk. The blood flows coming from VA-ECMO and LV are mixed: the saturation is decreased in the descending aorta and allows to detect easier Harlequin syndrome. Asterisk: The mixing point according to LV function. E. Screenshot of two peripheral oxygen saturations (right hand and lower limb) associated with invasive arterial pressure during 2 h. The represented curves are not the real-time data but the trends. Before LV recovery (a) the arterial pulse pressure is decreased (i.e., the stroke volume of LV is decreased (white asterisk) and the peripheral oxygen saturation of lower limb is increased (white arrow) at the same level of the saturation of right upper limb (white dotted arrow). After LF recovery (b), the arterial pulse pressure is increased (pink asterisk) with a lowered oxygen saturation in the lower limb (pink arrow), whereas the oxygen saturation remained normal in the right upper limb (pink dotted arrow)

The diagnosis of this HS must be suspected (and therefore prevented) with clinical, biological, blood gas and echocardiographic signs (Additional file 1: Table S1). To decrease the risk of severe cardiac failure, the HS should be evoked in all VA-ECMO patients. Unexpected EKG changes or recurrent signs of myocardial injury must be considered as very significant of it.

The best prevention of HS is to carefully consider VA-ECMO indications. Moreover, to prevent and/or treat a HS, several options can be proposed in function of LV function (Additional file 1: Table S1). First, improving the pulmonary function by optimizing respiratory pressures, inspired fraction of oxygen and using prone positioning. These strategies could improve the oxygen content of the blood flow ejected by LV. According to guidelines, this ventilation must remain protective with a limited driving pressure in order to avoid volo-barotrauma to the lung. Secondly, if the LV function is sufficient, the withdrawal of the VA-ECMO should be discussed with a conversion to VV-ECMO. Thirdly, if the patient is not weanable, the increase in VA-ECMO blood flow could be a possibility: the arterialized blood flow from VA-ECMO could improve the oxygenation in the coronary arteries but this strategy could impair the LV function by increasing LV afterload, risk of acute pulmonary edema and at the end could also impair the coronary perfusion pressure. Fourthly, hypothermia could be a strategy to decrease the cardiac outflow and the myocardial oxygen consumption. Finally, adding a re-injection cannula in the femoral vein to offer a hybrid veno-arterio-venous ECMO will help to ensure coronary oxygenation by potentially increasing transpulmonary blood flow and oxygen content of pulmonary blood at the cost of recirculation [5].

In conclusion, HS must be suspected and prevented in all VA-ECMO patients in order to avoid a prolongated or a secondary/recurrent cardiac dysfunction.

## Supplementary Information

**Additional file 1.** Diagnosis elements during the successive stages in the Harlequin syndrome and elements of discussion for ECMO management.

**Additional file 2.** Blood gas according to sample site.

## Data Availability

The datasets used and/or analyzed during the current study are available from the corresponding author on reasonable request.
