# Supplementary material for: Hidden Harlequin syndrome in neonatal and pediatric VA-ECMO
Source: Crit Care. 2022 May 20;26:146. doi: 10.1186/s13054-022-04017-w (PMC9121566; doi:10.1186/s13054-022-04017-w)
Supplement: Supplementary file 1 — Additional file 1. Diagnosis elements during the successive stages in the Harlequin syndrome and elements of discussion for ECMO management. [file 13054_2022_4017_MOESM1_ESM.docx]

| **Left ventricular function on VA ECMO** | **Saturation upper limb** | **Saturation lower limb** | **Arterial Pulse pressure** | **Troponin** | **Echocardiography** | **How to manage** |
| --- | --- | --- | --- | --- | --- | --- |
| **Profound failure** | Normal | Normal | Absent or severely impaired | Increased | 1. TVI low | 1. Mechanical ventilation optimizing or prone positioning |
|  |  |  |  |  | 1. No opening aortic valve | 1. No weaning VA-ECMO |
|  |  |  |  |  | 1. Color Doppler= VA-ECMO blood flow close to aortic valve | 1. VA-ECMO blood flow optimizing |
|  |  |  |  |  |  | 1. Discuss VAV-ECMO |
|  |  |  |  |  |  | 1. Discuss mild hypothermia |
| **Severe to moderate failure** | Normal | Normal | Partially restored | Increased | 1. TVI increases | 1. Mechanical ventilation optimizing or prone positioning |
|  |  |  |  |  | 1. Opening aortic valve | 1. Try to weaning VA-ECMO |
|  |  |  |  |  | 1. Color Doppler= mixing point into the ascending aorta | 1. Optimizing Inotropic drugs |
|  |  |  |  |  |  | 1. Discuss VV- or VAV-ECMO |
|  |  |  |  |  |  | 1. Discuss mild hypothermia |
| **Subnormal** | Normal or Decrease | Decrease | Subnormal | Low or increased in case of secondary LV failure | 1. TVI subnormal | 1. Mechanical ventilation optimizing or prone positioning |
|  |  |  |  |  | 1. Opening aortic valve | 1. Weaning VA-ECMO +++ |
|  |  |  |  |  | 1. Color Doppler= mixing point after the beginning of brachio-cephalic trunk | 1. Optimizing Inotropic drugs |
|  |  |  |  |  |  | 1. Discuss VV ECMO |

**Supplemental Table 1: Diagnosis elements during the successive stages in the Harlequin syndrome and elements of discussion for ECMO management**

*LV, Left ventricle; TVI, The aortic velocity–time integral; VA, Veno-arterial; VAV, Veno-arterio-venous; VV, Veno-venous; ECMO, Extracorporeal membrane of oxygenation*
