# Supplementary material for: Hidden Harlequin syndrome in neonatal and pediatric VA-ECMO
Source: Crit Care. 2022 May 20;26:146. doi: 10.1186/s13054-022-04017-w (PMC9121566; doi:10.1186/s13054-022-04017-w)
Supplement: Supplementary file 2 — Additional file 2. Blood gas according to sample site. [file 13054_2022_4017_MOESM2_ESM.docx]

**Supplemental Table 2: Blood gas according to sample site**

| Blood gas values | Sampling Site | |
| --- | --- | --- |
|  | **Outlet of the ECMO oxygenator** | **Femoral arterial blood gas** |
| pH | 7.45 | 7.44 |
| PaCO2 (mmHg) | 57 | 59 |
| PaO2 (mmHg) | 487 | 66 |
| Lactate (mmol/L) | 1.8 | 1.7 |
